# Effectiveness of community-based peer support for mothers to improve their breastfeeding practices: A systematic review and meta-analysis

**DOI:** 10.1371/journal.pone.0177434

**Published:** 2017-05-16

**Authors:** Prakash Shakya, Mika Kondo Kunieda, Momoko Koyama, Sarju Sing Rai, Moe Miyaguchi, Sumi Dhakal, Su Sandy, Bruno Fokas Sunguya, Masamine Jimba

**Affiliations:** 1 Department of Community and Global Health, Graduate School of Medicine, The University of Tokyo, Tokyo, Japan; 2 School of Public Health and Social Sciences, Muhimbili University of Health and Allied Sciences, Dar es Salaam, Tanzania; Cardiff University, UNITED KINGDOM

## Abstract

Breastfeeding is associated with reduced mortality in children aged less than 5 years. We conducted a systematic review and meta-analysis (registered as PROSPERO 2015: CRD42015019105) to examine the effectiveness of community-based peer support for mothers on their breastfeeding practices as compared to mothers who have not received such a support.

**Methods:**

We searched for evidence regarding community-based peer support for mothers in databases, such as PubMed/MEDLINE, the Cochrane Library, CINAHL, Web of Science, SocINDEX, and PsycINFO. We selected three outcome variables for breastfeeding practices, namely, exclusive breastfeeding duration, breastfeeding within the first hour of life, and prelacteal feeding. We conducted meta-analyses of the included randomized controlled trials and quasi-experimental studies.

**Results:**

For our review, we selected 47 articles for synthesis out of 1,855 retrieved articles. In low- and middle-income countries, compared to usual care, community-based peer support increased exclusive breastfeeding at 3 months (RR: 1.90, 95% CI: 1.62–2.22), at 5 months (RR: 9.55, 95% CI: 6.65–13.70) and at 6 months (RR: 3.53, 95% CI: 2.49–5.00). In high-income countries, compared to usual care, peer support increased exclusive breastfeeding at 3 months (RR: 2.61, 95% CI: 1.15–5.95). In low- and middle-income countries, compared to usual care, peer support increased the initiation of breastfeeding within the first hour of life (RR: 1.51, 95% CI: 1.04–2.21) and decreased the risk of prelacteal feeding (RR: 0.38, 95% CI: 0.33–0.45).

**Conclusions:**

Community-based peer support for mothers is effective in increasing the duration of exclusive breastfeeding, particularly for infants aged 3–6 months in low- and middle-income countries. Such support also encourages mothers to initiate breastfeeding early and prevents newborn prelacteal feeding.

## Introduction

Appropriate breastfeeding practices improve child survival, health, and development [1]. Globally, about 1.4 million child deaths are attributed to suboptimal breastfeeding [2]. Exclusive breastfeeding (EBF) during the first 6 months of life can reduce child mortality by preventing diarrhea and pneumonia [2]. Moreover, when breastfeeding is initiated early, it can reduce neonatal mortality [3, 4]. Therefore, the World Health Organization (WHO) recommends EBF until 6 months of age and breastfeeding initiation within the first hour of life.

Even though these recommendations were issued more than 25 years ago, breastfeeding rates still remain far below set targets in many countries [5]. The EBF rates are well below 50% in most countries [1]. For example, only 37% of infants living in low- and middle-income countries (LMICs), are exclusively breastfed [1]. Infants living in high-income countries have even shorter breastfeeding duration. Globally, only about 50% of neonates are breastfed within their first hour of life [1]. Professional health workers can help improve breastfeeding practices; however, in resource-limited settings, the lack of such health workers hinders effective breastfeeding promotion. Therefore, we have to depend on mothers themselves, as they are the primary caregivers. When mothers participate in groups for social activities or receive one-on-one counseling from another mother in the community, they can communicate with each other and exchange knowledge among themselves. Such one-on-one or group peer support for mothers allow them to support each other, helps in decision making and subsequently empowers them [6]. Thus, these interventions have potential to improve breastfeeding practices and child wellbeing [7]. Such interventions have therefore been considered as sustainable alternatives to counseling in primary health care settings [8]. These are potentially lower cost interventions compared to those provided by professional health care workers [9].

Evidence is available on how effective peer support interventions for mothers (one-on-one or in a group) can be in improving breastfeeding practices, but the results remain inconsistent [10, 11]. We conducted this systematic review and meta-analysis to collate and summarize such evidence. We aimed to examine the effectiveness of community-based peer support for mothers on their breastfeeding practices as compared to mothers who have not received such a support.

## Methods

We developed and followed a standard systematic review protocol (systematic review registration-PROSPERO 2015: CRD42015019105) in accordance with the PRISMA statement [12](S1 Table). We established four review teams, each one comprised of two researchers who worked independently to search, extract data, review, and assess the quality of the studies. We settled any disagreements among the review team via discussions until reaching a unanimous decision.

### Eligibility criteria

#### Types of participants

We reviewed studies involving mothers of children less than five years of age.

#### Types of interventions

Studies were eligible if they focused on one-on-one and/or group peer support for mothers, including peer nutrition counseling, shared decision making, grandmothers/elders-to-mother nutrition counseling. We also included studies that had nutrition-focused participatory interventions involving mothers themselves as key drivers. We excluded studies that included top-down nutrition interventions, such as the distribution of ready-to-use therapeutic foods, supplemental blanket feeding, and cash transfer.

#### Types of outcomes

The primary outcome of the original study protocol was the child's nutritional status (assessed by being underweight, the presence of stunting and/or wasting), and the secondary outcome was child feeding practices (including breastfeeding and other feeding practices). However, we did not find a sufficient number of eligible studies that analyzed the role of community-based interventions on child nutritional status and feeding outcomes, such as complementary feeding, feeding frequency, and dietary diversity. Therefore, we focused on breastfeeding practices. We utilized WHO definition of EBF and defined EBF duration as the period when an infant receives only breast milk without any other liquids or solids, including water and expressed breast milk. We included breastfeeding initiation within the first hour of life and prelacteal feeding as post-hoc additional outcomes. We defined prelacteal feeding as any food or fluid provided to a newborn before initiation of breastfeeding[13].

#### Types of studies

We selected studies that included randomized controlled trials (RCTs), quasi-experimental, cohort, cross-sectional, and other comparative observational studies.

### Search strategy

We conducted the search process in six medical databases. These were as follows. PubMed/MEDLINE, the Cochrane Library, CINAHL, Web of Science, SocINDEX, and PsycINFO. We used the following search strategy for the PubMed/Medline database: (((((((((group, women's[MeSH Terms]) OR groups, women's[MeSH Terms]) OR club, mothers'[MeSH Terms]) OR mothers[MeSH Terms]) OR clubs, mothers[MeSH Terms]) OR mother's group) OR mother's groups)) AND (((((((((empowerment[MeSH Terms]) OR community based participatory research[MeSH Terms]) OR participatory research, community based[MeSH Terms]) OR participatory intervention) OR peer groups[MeSH Terms]) OR peer group[MeSH Terms]) OR counseling[MeSH Terms]) OR peer counseling) OR shared decision making[MeSH Terms])) AND (((((((((((nutritional status[MeSH Terms]) OR sciences, child nutritional[MeSH Terms]) OR nutritional index[MeSH Terms]) OR anthropometry[MeSH Terms]) OR nutrition) OR feeding pattern[MeSH Terms]) OR feeding behavior[MeSH Terms]) OR feeding behaviors[MeSH Terms]) OR feeding practice) OR complementary feeding[MeSH Terms]) OR breastfeeding[MeSH Terms]) AND (("1978"[Date—Publication]: "2015"[Date—Publication])). Then we used similar keywords to search in the other databases selected.

We also searched international organization databases such as the World Bank, UNICEF, and the WHO databases. We used the cited references of retrieved articles to hand search. We limited all the evidence to abstracts published in the English language between 1978 to the end of March 2015. The year 1978 was selected because the Alma Ata declaration was made in this year, and it emphasized community participation as an important primary health care component.

### Study selection

We allocated the searched and selected articles equally among the four review teams. Then, two researchers in each review team independently performed the eligibility assessment in a blinded standardized manner.

### Assessment of risk of bias

The two researchers in each review team independently assessed the risk of bias for the studies included. We used the risk of bias assessment tool developed by Cochrane collaboration for assessing RCTs and quasi-experimental studies [14]. For observational studies, we used "Risk of Bias Assessment Tool for Nonrandomized Studies" (RoBANS) criteria [15]. We evaluated the risk of bias for RCTs and quasi-experimental studies based on the following criteria: sequence generation/randomization, allocation concealment, blinding of participants, blinding of outcome assessment, incomplete outcome data, and selective outcome reporting and other potential biases. For non-RCTs, risk of bias was assessed based on criteria, such as the selection of participants, consideration of confounding variables, appropriate measurement of exposure, blinding of outcome assessments, completeness of outcome data, and how outcomes were reported. We looked for an explanation for each criterion (of Cochrane tool or RoBANS) in the studies and judged them as low, unclear, or high risk of bias. Any disagreements were settled through discussion and unanimous agreement among the reviewers.

### Data collection

The two researchers in each review team independently extracted the data and entered it in a standardized data extraction matrix. The following information was retrieved during data extraction: study location, design, participants, type of intervention, training of the mothers, the presence of professional help, the comparison group, and reported outcomes of interest.

### Meta-analysis

A meta-analysis of RCTs and quasi-experimental studies using Review Manager (Revman) 5.1 software was conducted. We used a random effect model of meta-analysis throughout and presented results as risk ratios (RRs) with their 95% confidence intervals (CI).

We conducted the meta-analyses by stratifying the studies into LMICs and high-income countries as defined by the World Bank [16]. We also conducted subgroup analyses by EBF duration. The strategy for these analyses was planned during the data extraction stage. We estimated the percentage of variability across studies attributable to heterogeneity with the I^2^ statistic. We also performed sensitivity analysis, where appropriate, to evaluate whether pooled effect sizes were robust across the components of risk of bias.

We included 22 studies in the meta-analyses of different outcomes. We excluded two studies that assessed multifaceted interventions. Such multifaceted interventions make it difficult to isolate and attribute the effect of peer support for mothers [17, 18]. We also excluded one study, owing to the lack of a comparison group [19].

### Ethics statement

This study was a review and dealt only with data published in the selected studies. We had no direct access to the original data used in any of those studies. Therefore, no ethical approval was warranted for this review.

## Results

### General characteristics of the selected studies

**Fig 1:**
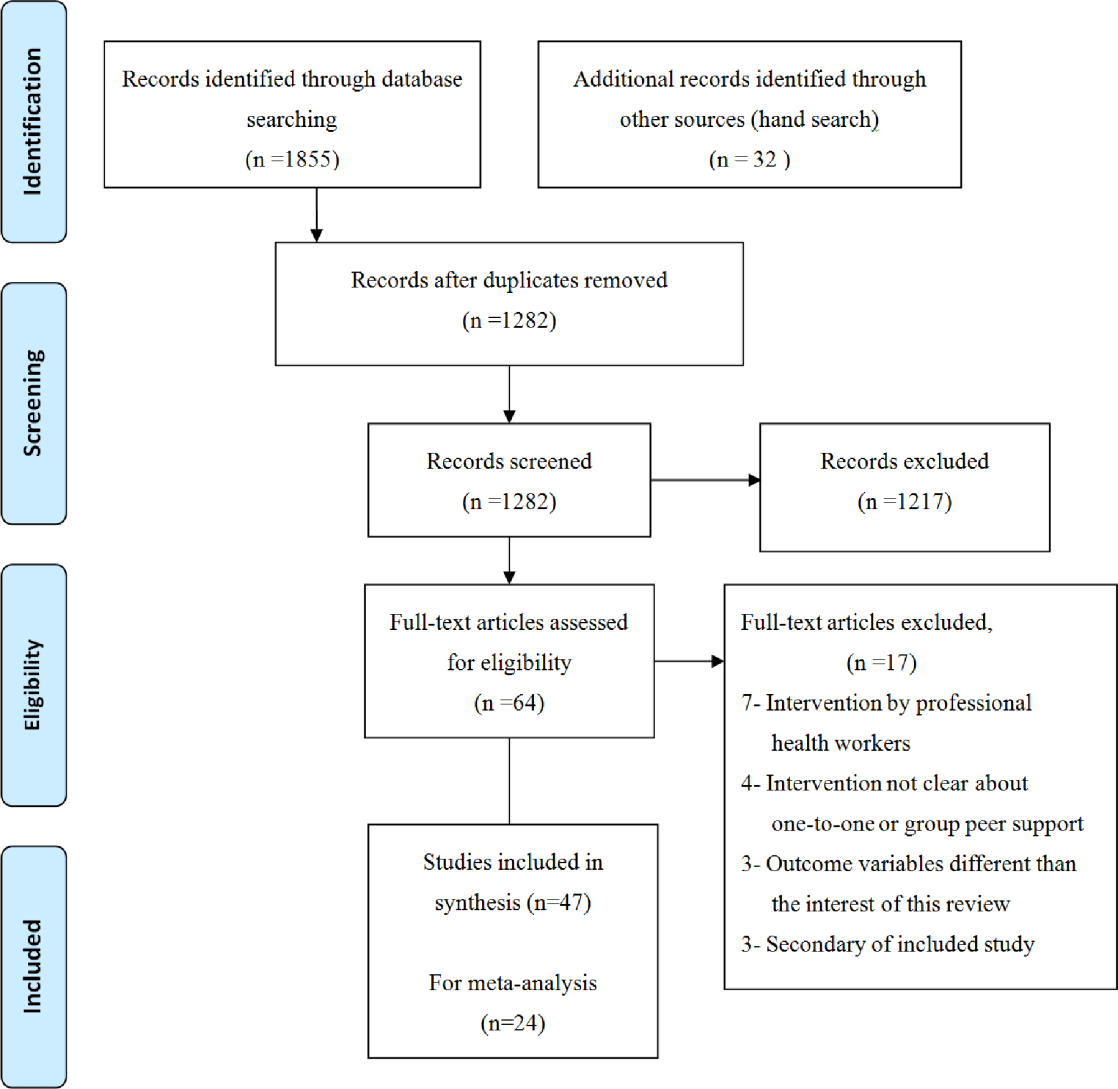
Flow diagram of included and excluded studies.

Fig 1 demonstrates the selection process and search results. Following an extensive literature search, 1,855 articles were retrieved. After removing the duplicates, 64 articles were eligible for full text review. Among them, 17 articles did not meet the inclusion criteria (S2 Table). Finally, a total of 47 articles were included in the systematic review.

Table 1 presents a summary of the descriptive characteristics and the results of 47 studies included in this review. Of them, 27 studies were conducted in high-income countries and 20 in LMICs. One study was conducted in three LMICs of sub-Saharan Africa (Burkina Faso, South Africa, and Uganda) [20]. Of 47 studies, 20 were conducted in Northern America (USA and Canada), three in Latin America (Brazil and Mexico), three in Western Europe (UK), one in Western Asia (Turkey), nine in Southern Asia (India and Bangladesh), one in Eastern Asia (China), one in South-Eastern Asia (Philippines), three in Eastern Africa (Uganda, Malawi and Kenya), one in Western Africa (Burkina Faso), and one in Southern Africa (South Africa). In total, 22 studies were conducted in urban settings, 19 in rural settings, one in a mixed setting, and seven did not specify their settings. Of the total 47 studies, 28 were RCTs, 13 were quasi-experimental studies, and six were observational studies. Two articles presented the results of the same RCT but with different outcome variables [21, 22].

**Table 1:** Characteristics of included studies.

Study	Area	Study design	Study population	Intervention	Training	Comparison	Reported Outcomes
Acharya, 2015 India	Rural	Cluster randomized controlled trial (RCT)	Expectant mothers	Raise awareness on safe pregnancy and neonatal care + Home Visits + Mass Media + Mothers groups meetings	Community-based Accredited Social Health Activists (ASHAs): trained in interpersonal communication and group facilitation for 6–9 months	Raise awareness on safe pregnancy and neonatal care + home visits + mass media	a) Initiation of Breastfeeding (BF) within 1 hour: Intervention group (IG) 42.37% and Control group (CG) 29.10% b) Exclusive Breastfeeding (EBF) at 6 months: IG 20.51% and CG: 11.70%
Agrasada, 2005 Philippines	Urban	RCT	Primiparous mother intending to breastfeed a low birth weight (<2500 g) singleton born between 37 and 42 weeks	Home visits by peer counselor mothers at days 3–5, 7–10, 21, 1.5 months followed by monthly visits up to 5.5 months post delivery	40 hours of training from certified lactation counselor	Usual care	EBF at 6 months- IG (breastfeeding counseled mothers) 44%, IG (child care counseled mothers) 7% and CG 0%
Ahluwalia, 2000 USA	Not specified	Mixed method (Secondary Survey data and focus group discussions (FGDs))	Mother-infant pairs enrolled in public health clinics for Women Infants and Children (WIC) program	Former WIC participants recruited to provide support and encouragement to current WIC participants	Details not available	No comparison group	a) Any initiation of BF: Before intervention 39.5% and after intervention 50.4% b) Any BF at 8 weeks: Before intervention 18.3% and after intervention 19.4%
Aksu, 2011 Turkey	Urban	RCT	Singleton healthy infant > = 37 weeks, infants <2500 g, Apgar < = 7 and congenital anomalies excluded	Home visit by two supporter mothers 3 days after the birth and give breastfeeding education on day 3 after delivery	18 hour WHO/UNICEF breastfeeding support course	Breastfeeding education from nurses	a) EBF at 2 weeks: IG 67% and CG 40% b) EBF at 1.5 months: IG 60% and CG 33% c) EBF at 6 months: IG 43% and CG 23% d) Initiation of BF within 1 hour: IG 90% and CG 93.3%
Anderson, 2005 USA	Urban	RCT	Pregnancy <32 weeks, predominantly Hispanic, healthy full term singleton baby	EBF peer counseling support offering 3 prenatal home visits, daily perinatal visits, 9 postpartum home visits, and telephone counseling	2 peer mothers trained by Lactation Consultant for 2 weeks using 40 hour WHO/UNICEF Training manual	Usual care, conventional breastfeeding education	a) Non-exclusive BF at 1 month: IG 65.1% and CG 91.6 b) Non-exclusive BF at 2 months: IG 71.4% and CG 71.4% c) Non-exclusive BF at 3 months: IG 73.0% and CG 97.2%
Arifeen, 2009 Bangladesh	Rural	cluster RCT	Pregnant women in 20 government health facilities	IMCI home visits and mother's group meetings by community nutrition promoters and village health workers	IMCI 2 days training course for feeding practices	Usual care	Complementary food with continued BF up to 2 years: 6–9 months child, IG 67.6% and CG 57.2%
Arlotti, 1998 USA	Not specified	Quasi-experimental	Prenatal and postpartum mothers of age 15–36 years old who are enrolled in WIC program	Peer counselor mothers contacted the participants within a few days after delivery, and 2 weeks, 1month, 2 month, and 3 months after delivery. Contacts were made by telephone, letter or in person at the WIC office	20-hr training program in breastfeeding and communication skills, developed by La Leche League International and administered by the breastfeeding peer counselor coordinator for WIC in the county	Usual care	Mean rates of EBF for IG: 53% at 2 weeks, 40% at 1 month, 33% at 2 months, and 17% at 3 months and for CG: 17% at 2 weeks, 27% at 1 month, 13% at 2 months, and 6% at 3 months
Bhandari, 2003 India	Rural	RCT	Infants born within 9 months of the start of the intervention	Local village based health workers (Anganwadi workers) conducted monthly home visits and assessed an infant’s feeding practices, identified difficulties, and provided information on the benefits of exclusive breastfeeding	A 3 days training course based on an adaptation of the "Integrated Management of Childhood Illnesses Training Manual on Breastfeeding Counselling"	Usual care	a) Initiation of BF within 3 hours: IG 49.7% and CG 23.7% b) Prelacteal feeding: IG 31.0% and CG 74.7% e) EBF at 3 months: IG 78.8% and CG 47.8% c) EBF at 4 months: IG 68.7% and CG 11.9% d) EBF at 5 months: IG 49.3% and CG 6.1% e) EBF at 6 months: IG 41.6% and CG 3.9% f) Mean duration of EBF: IG 122 days and CG 41 days
Bhandari, 2004 India	Rural	RCT	Infants born within 9 months of the start of the intervention	Local village health workers (Anganwadi workers) conducted monthly home visits for newborns until aged 12 months	A 3 days training based on "Integrated Management of Childhood Illness manual on nutrition counseling"	Usual care	Feeding frequency of complementary food at 9 months of age: IG 4.4 times/24h and CG 3.9 times/24h f) Feeding frequency of complementary food at 18 months of age: IG 5.9 times/24h and CG 5.4 times/24h
Brown, 2011 UK	Not specified	Mixed method	Mothers with at least one child between 6–24 months of age	Not an intervention study but association with attending a breastfeeding support group was examined retrospectively	Details not available	Those who did not attend breastfeeding support group	Breastfeeding for at least six months was positively associated with attending a breastfeeding support group
Campbell, 2014 USA	Not specified	Retrospective cross-sectional	Singleton women who had not previously breastfed	Support by women, infant and children (WIC) peer counselors who counseled about breastfeeding, visit women in hospitals for mother to mother support	20 hour training for WIC peer counsellors who also have been recipients of WIC services themselves previously	Those who did not have WIC peer counselor	Mothers who had peer counselor support during pregnancy (OR 1.50, 95% CI 1.24–1.82, p < .001) or after delivery(OR 1.93, 95% CI 1.60–2.32, p < .001) were significantly more likely to initiate BF than mothers who did not have a peer counselor support
Chapman, 2004 USA	Urban	RCT	Predominantly Hispanic women with <27 weeks’ gestation	Routine breastfeeding, education plus peer counselling: ≥1antenatal home visit, daily in hospital visits, and ≥3 postnatal home visits	Trained in breastfeeding management + in-service training, close monitoring, 1 hour/month continuing education	Routine breastfeeding education only	a) Not Initiating of BF: IG 8.9% and CG 22.7% b) Not any BF at 1 month: IG 35.7% and CG 49.3% c) Not any BF at 3 months: IG 55.6% and CG 70.8% d) Not any BF at 6 months: RR 0.94; 95% CI 0.79–1.11
Chola, 2013 Uganda	Rural	Data from a previous RCT (PROMISE-EBF study)	Singleton birth without congenital malformation	Home visits by peer counselor mothers- one during antenatal and at least four during postnatal period at weeks 1, 4, 7, and 10	Trained for 1 week on WHO Breastfeeding support, HIV and Infant feeding course	Usual care	Children in intervention group (hazard ratio = 0.33, 95% CI 0.26–0.42) and rural areas(hazard ratio = 0.79, 95% CI 0.63–0.99) had a lower risk of EBF/PBF (predominant breastfeeding) cessation.
Dennis, 2002 Canada	Not specified	RCT	Primiparous mother initiated breastfeeding, aged at least 16, singleton birth at37 weeks or onwards	Telephone-based peer support initiated within 48 hours of hospital discharge	A 2.5 hour orientation session focusing on peer telephone support, referral skills and problem solving skills. A handbook was also given to all peer volunteers	Usual care and conventional postnatal support	a) EBF at 1 month: IG 74.2% and CG 62.9% b) EBF at 2 months: IG 56.8% and CG 40.3% c) EBF at 3 months: IG 62.9% and CG: 54.8%
Di Meglio, 2010 USA	Not specified	RCT	Mothers with infants of ≥36 weeks’ gestation, birth weight >2000 g	Telephone-based peer support at 2,4, and 7 days post discharge and then, at 2,3, 4 and 5 weeks post-discharge	Breastfeeding peer counselor training program consisting of 10, 2-hour sessions developed and delivered by La Leche League leaders	Usual care	a) Any breastfeeding duration (median): IG 75 days and CG 35 days b) Hazard ratio of breastfeeding cessation: 0.71, 95% CI 0.39–1.30, p = 0.26
Edwards, 2013 USA	Urban	RCT	Pregnant African American women < 34 weeks gestation, under 21 years of age	Doulas (specialized home visitors trained as childbirth educators and lactation counselors provided an average of 10 prenatal and 12 postpartum home visits	An intensive 20-week training course provided by the Chicago Health Connection and a 10 week breastfeeding peer counselor training program	Usual prenatal health care services	Intervention group mothers attempted breastfeeding at a higher rate than control group mothers (64% vs 50%; P = 0.02) and were more likely to breastfeed more than 6 weeks (29% vs 17%; P = 0.04)
Graffy, 2004 UK	Urban	RCT	Women in their 28-36^th^weeks of pregnancy	Antenatal and postnatal counseling support by peer counselors by telephone or further home visits	Had undertaken training on counseling mothers (details not available)	Usual care	a) EBF at 6 weeks: IG 30.6% and CG 25.6% b) Any BF at 6 weeks: IG 64.8% and CG 63.4% c) Any BF at 4 months: IG 46.1% and CG 42.2%
Gross, 1998 USA	Urban	Cluster RCT	African American women with singleton pregnancy of less than 24 weeks gestation	Peer counselor mothers conducted group support sessions on infant feeding during prenatal period, follow-up to 16weeks postpartum	A 5-week training program that was adapted from the District of Columbia WIC. The training consisted of ten 2.5 hour sessions	Usual WIC services including nutrition education and breast feeding promotion activities	Any BF at16 weeks: IG (video +peer counseling) 40%, IG (video only) 48%, IG (peer counseling only) 52% and CG 0%
Gross, 2009 USA	Urban	Cross-sectional	Pregnant or postpartum women and children under 5 years of age	Peer counselor (PC) mothers contacted the pregnant participants at 1 month, 2 weeks prior to the delivery date, and around the time of delivery, For postpartum participants contacts every week for infant's first 4 weeks, then at 3, 6, 9, and 12 months, and once a year until the child is weaned	Trained using a standard classroom curriculum, “Loving Support Through Peer Counseling.” It included 20 hours of classroom instruction	Standard care group (SCG)participants received breastfeeding information during the prenatal visit. Lactation group (LG) group participants receive the same breastfeeding education as the SCG group, but can have support from certified lactation consultant if requested	Any initiation of BF: PC 60.9%, LC 54.5% and SCG 47.3%
Guldan, 2000 China	Rural	Quasi-experimental	Mother and infant pair	Community-based pilot nutrition education intervention: Village nutrition educators made monthly visits to the pregnant women and the infants born	Three training sessions on growth monitoring, counseling on feeding and communication skills for each half to 1 day duration	Usual care	Overall BF rate: IG 83% and CG 75%
Haider, 2000 Bangladesh	Urban	RCT	Women aged 16–35 years, infants excluded with birth weight < 1.8 kg or congenital anomalies	Home visits by peer counselor mothers at 48 hour of delivery, one on day 5, one during days 10–14, and every two weeks for 2–5 months	40 hours of training with WHO breastfeeding support and King's book	Usual care	a) Initiation of BF within 1 hour: IG 63.7% and CG 15.4% b) Prelacteal feeding: 31.3% and CG 89.1% c) EBF at 5 months: 88.6% and CG 6.0%
Hoddinott, 2006 UK	Rural	Quasi-experimental	Mother and infant pair	Mothers group meetings and one-on- one peer coaching on breastfeeding	Two seminars of 2 hours duration	Usual care	Significant increase in any BF at 2 weeks for intervention group from 34.3% to 41.1% (95% CI 1.2–12.4)
Ingram, 2013 UK	Urban	Mixed method(Online survey, semi-structured interviews and FGDs)	Mothers in 12 areas of low breastfeeding prevalence in the city	Breastfeeding peer support service for mothers with one antenatal visit and postnatal contact at 48 hours after coming home which continued for 2 weeks	Peer supporters' training based on La Leche League which comprised 10 sessions of 2.5 hours each initially, with extra Safeguarding and Lone Working sessions added later	Compared with rest of the city (Bristol) other than 12 targeted peer support wards	a)Any BF at 8 weeks: IG- 37.4% (2010), 38.6% (2011) and CG- 69.5% (2010), 68.6% (2011) b)Initiation of BF: IG- 64.6% (2010), 66.7% (2011) and CG- 88.7% (2010), 88.9% (2011) c) EBF at 8 weeks: IG- 25.3% (2010), 27.20% (2011) and CG- 48.3% (2010), 47.7% (2011)
Jolly, 2012 UK	Urban	Cluster RCT	Pregnant women aged 16–35 years, no more than three living children or parity 5, infants excluded with birth weight < 1.8 kg or congenital anomalies	Peer support workers gave antenatal (2 support sessions at home and clinic) and postnatal (clinic, home, telephone) support	Trained by the breast-feeding personnel over 8 weeks	Usual care	a) EBF at 6 weeks: IG 38.5% and CG 40.9% b) EBF at 6 months: IG 17.8% and CG 19.6%
Khan, 2013 Bangladesh	Rural	RCT	Pregnant women with a viable fetus<14 weeks gestation	Home based counseling on EBF by female workers recruited from the local community in eight visits: two during the last trimester of pregnancy, one within 7 days of delivery and five at monthly intervals up to 6 months after delivery	Trained using a 40 hour WHO/UNICEF Breastfeeding Counseling Training module in local language	Usual/standard health messages (UHM) delivered by the regular healthcare staff during postnatal clinic visits	a) EBF at 4 months: IG 68.0% and CG 46.0% b) EBF at 6 months: IG 15.0% and CG 4.0% c) Mean duration of EBF: IG 111 days and CG 76 days
Kisten, 1994 USA	Urban	Quasi- experimental	Low income women who delivered infants at Cook county hospital, Chicago	Telephone counseling on breastfeeding practices by peer counselor mothers at least twice a week after delivery until breastfeeding was established and every one to two weeks for the next two months	One 12–16 hour training for peer counselors by a registered nurse. The content of training sessions included breastfeeding promotion, breastfeeding management, nutrition etc.	Women who requested counselors but, owing to inadequatenumber of trained counselors, did not have a counselor	a) Initiation of BF at discharge: IG 93% and CG 70% b) EBF at 1.5 months: IG 44% and CG 16% c) EBF at 3 months: IG 29% and CG 7% d) Mean no. of weeks of EBF: IG 8 and CG 4 e) Mean number of weeks of any BF: IG 15 and CG 8
Kushwaha, 2014 India	Rural	Quasi-experimental	Mother-infant pairs	Mothers support groups (MSG) conducted home visits. It comprised of 3–4 members, namely, a traditional birth attendant, an experienced mother, and a community health/nutrition worker. Home visits: 10 visits in the first 6 months, 6 visits in the next 6 months and 3 visits during the 2nd year	Training in a 3day workshop using the International Baby Food Action Network (IBFAN)/WHO/UNICEF/Breast Feeding Promotion Network of India (BPNI) 3-in-1 training modules	No control group Before-after study Outcomes measured at baseline/pre-intervention in 2006 (T0) compared with post-intervention in 2008 (T1) and 2011 (T2)	a)Initiation of BF within 1 hour: T0 11%, T1 71% and T2 62% b) No use of prelacteal feed: T0 33%, T1 85% and T2 95% c) EBF at 6 months: T0 7%, T1 50% and T3 60% d) Initiation of complementary feeding (6–8 months): T0 54%, T1 85% and 96% e) Complementary foods along with continued BF up to 2 years: T0 4.5%, T1 36% and T2 42%
Le Roux, 2011 South Africa	Urban	RCT	Mothers and their malnourished children under the age of 6 years	Mentor Mothers (MM) nominated from local community conducted home visits throughout one year	Four phases of training by Philani outreach supervisors which included sessions on nutrition, basic child health, weighing babies, recognizing danger signs etc.	No intervention visits by MMs	The quadratic time trend showed underweight/ WAZ score in intervention group increased linearly over one year compared to the control group (slope difference = 0.018, t = 2.25, df = 5176, p = 0.02)
Leite, 2005 Brazil	Rural	RCT	Singleton healthy infant <3000g	Home visit by peer counselor mothers on the 5th, 15t, 30th, 60th, 90th and 120th days post delivery	20 hours training course on breastfeeding support	Usual care	EBF at 4 months: IG 24.7% and CG 19.4%
Lewycka, 2013 Malawi	Rural	Cluster RCT	A cohort of women of childbearing age	Three intervention groups: Women’s group (WG) + volunteer peer counselling (VPC), WG only, and VPC only. WG was conducted by a cluster peer facilitator through a community mobilization action cycle of 20 meetings in four phases.VPC consisted of home visits by peer counselors in the third trimester, in the week after birth, and at 1 month, 3 months, and 5 months	WG facilitators were trained over 11 days, with refresher training every 4 months. VPC were trained for 5 days and annual refresher training. They also attended monthly meetings	No intervention (WG or VPC)	a) Initiation of BF within 1 hour: WG+VPC -72%, WG only-83%, VPC only- 72% and control group- 77% b) EBF at 6 months: WG+VPC- 26%, WG only- 10%, VPC only-14% and control group- 7%
Long, 1995 USA	Rural	Quasi-experimental study with historical control	Native American pregnant women who met the enrollment criteria for the WIC program (family income no more than 185 percent of the poverty level)	Peer counselors contacted pregnant and breastfeeding women by telephone, home visits, and/or clinic visits prenatally and at 1, 2, 4 to 6 weeks postpartum	12-hour breastfeeding peer counseling training program developed by the Utah WIC program	Historical controls from retrospective data of all women enrolled in the WIC program at the Salt lake city Indian Health care center who gave birth between Jan. 1991 and Jan. 1992	a) Any Initiation of BF:- IG 84% and CG 70% b) Any BF at 1 month: IG 71% and CG 57% c) Any BF at 2 months: IG 55% and CG 41% d) Any BF at 3 months: IG 49% and CG 36% e) Any BF at 6 month: IG 21% and CG 31%
Lovera, 2010 USA	Urban	Cohort	Cohorts of Hispanic couples aged more than 18 years participating in WIC program: Fathers participated in the pilot Peer Dad Program and/or mothers participated in the Peer Counselor Program during the same time period	Couples participated in peer counseling and peer dad counseling program. Individual counseling by telephone or in person provided by peer dads prenatally and postnatally to other WIC fathers	Details not available	Mothers who participated in peer counseling only	a) Any BF less than 3 months: IG 17.8% and CG 25.3% b) Any BF 3–6 months: IG 18.8% and CG 20.2% c) Any BF 6–12 months: 32.7% and CG 24.2% d) Any BF for 12 months: IG 30.7% and CG 30.3%
McInnes, 2000 UK	Urban	Quasi-experimental	Women attending the local antenatal booking clinic	Peer counselor mothers "helpers" visited pregnant women four times; twice antenatally and twice postnatally to counsel on infant feeding and breastfeeding	Training to promote breastfeeding and support breastfeeding mothers	Usual care	EBF at 6 weeks: IG 8.3% and CG 5.3%
Merewood, 2006 USA	Rural	RCT	Mothers with otherwise healthy premature infant (26–37 weeks’ gestation)	Hospital and home based support by peer counselor mothers. Initial face to face contact within 72 hours while still in hospital then weekly contact for six weeks	Trained with 5-day BF course and at hospital on NICU, BF techniques, etc.	Usual care	Any BF at 12 weeks: odds ratio 2.81, 95% CI 1.11–7.14, P = 0.01
More, 2012 India	Urban	Cluster RCT	Live births in study area from 2006 to 2009	Women's group meetings facilitated by trained local women "sakhi". Meetings consisted of an action learning cycle with 7 phases in which they discussed perinatal experiences, improved their knowledge, and took local action	Details not available	Usual care	a) Initiation of BF within 24 hour: IG 82.7% and CG 82.4% b) EBF at 1 month: 70.4% and CG 66.7%
Morrow, 1999 Mexico	Urban	RCT	Pregnant women residing in the study area	Two intervention groups with different counseling frequencies, six visits (mid and late pregnancy and weeks 1, 2, 4 and 8) and three visits (late pregnancy and week 1 and 2) by peer counselor mothers	Trained and supervised by staff of La Leche League of Mexico and the physician study coordinator. It consisted of 1 week of classes, 2 months in lactation clinics and with mother-to mother support groups, and 1 day of observation, demonstration by visiting experts and practice for 6 months before trial	Usual care	a) Initiation of BF within few hours of birth: 6 Visit group 65.9%, 3 visit group 59.6% and CG 67.6% b) EBF at 3 months: 6 Visit group 67.0%, 3 visit group 50.0% and CG 12% c)Diarrheal episode: IG (combined): 12% and CG 26%
Muirhead, 2006 UK	Rural	RCT	Pregnant women at 28 weeks gestation	Home visit or phone call by peer supporter at least once during the antenatal period. Further antenatal support was provided if mother requested	Peer supporter training on breastfeeding, transferable skills, health, safety and confidentiality and relationship with patients and professionals for 2 full days and four evening sessions with regular follow-up sessions	Usual care	a) EBF at 6 weeks: IG 24.1% and CG 21.2% b) EBF at 2 months: IG 20.5% and CG 14.2% c) EBF at 4 months: IG 1.8% and CG 0.0%
Navarro, 2013 Brazil	Not specified	Quasi-experimental	Mother-child pair	Mothers group meetings every fifteen days according to protocols defined in ten educational meetings on health and nutrition during pregnancy and monthly home visits by community counselors. Also home visits were carried out fortnightly during the first month and half after child birth to support breastfeeding and newborn care	60-hour basic training facilitated by health workers previously trained in IMCI community component	Usual care	EBF at 6 months: IG 7.3% and CG 2.3%
Ochola, 2013Kenya	Urban	RCT	Pregnant HIV-negative women at 34–36 weeks gestation reporting at the health center for antenatal services	Home based intensive counseling group (HBICG) received seven counseling sessions at home by peer counselors, one prenatally and six postnatally. Facility based semi-intensive counselling group (FBSICG) received only one counselling session prenatally	40-hour training on breastfeeding and complementary feeding based on the WHO/UNICEF counselling course and a book, *Helping Mothers to Breastfeed*	Usual standard health and nutrition education offered at the Langata Health Centre by the health staff	a) EBF at 1 month- HBICG 87.0%, FBSICG 84.3% and control 72.0% b) EBF at 3 months- HBICG 61.4%, FBSICG 47.2% and control 36.8% c) EBF at 6 months- HBICG 23.9%, FBSICG 9.2% and control 5.6%
Pugh, 2002 USA	Urban	RCT	Low income predominantly minority women with infant	Usual breastfeeding support and supplementary visits from the community health nurse/peer counselor team which included daily visits during hospital stay period and home visits during weeks 1,2, and 4. Peer counselors also provided telephone support twice weekly through 8th week and weekly through 6th month	Details not available	Usual breastfeeding support which consisted of support from nurses, telephone assistance and one hospital visit by lactation consultant	a) EBF at 3 months: IG 45% and CG 25% b) EBF at 6 months: IG 30% and CG 15%
Rempel, 2012 Canada	Urban	Quasi-experimental	Expectant mothers	A 2-hour workshop style Peer-led class(PLC) was provided to the participants by two volunteer Breastfeeding Buddies (BB)	All BB were provided an 18-hour breastfeeding training course adapted from the WHO's breastfeeding course	A nurse-led class(NLC) was provided to give information about breastfeeding practices	PLC participants had intention to breastfeed longer than NLC participants, 9.6 months (SD 2.6) vs 7.4 months (SD 4.1)
Schafer, 1998 USA	Rural	Quasi-experimental	Rural low income pregnant and postpartum women who qualified for WIC program	One-on-one volunteer peer support provided by peer counselors during antenatal and postnatal period in home or WIC clinic. Information on breastfeeding and healthy diet was provided	9 hours of training from the project staff members on general nutrition, advantages of BF, basic management of BF, home visit skills, etc.	Usual care	a) Any initiation of BF: IG 82% and CG 31% b) Any BF at 2 weeks: IG 81% and CG 18% c) Any BF at 4 weeks: IG 56% and CG 10% d) Any BF at 8 weeks: IG 48% and CG 10% e) Any BF at 12 weeks: IG 43% and CG 10%
Shaw, 1999 USA	Rural	Quasi-experimental	Women between 6 weeks and 6 months postpartum and had registered ante partum for WIC program	Peer counselors provided counseling on breastfeeding via home visit, telephone or clinic visit	Peer counselors got individualized training by a nutrition educator and 56 hours of continuing education	Usual care	a) Any initiation of BF: IG 53% and CG 33% b) Any BF ≥ 6 weeks: IG 26% and CG 13%
Taveras, 2011 USA	Urban	Quasi-experimental	Mother-infant pairs who received their pediatric care at the 3 designated primary care offices	A 6-month, multifaceted intervention which consisted of brief focused negotiation by pediatric primary care providers, four individualized coaching and motivational counseling telephone calls by a study health educator, and four group parenting skills training workshops which also included promotion of peer support +usual care by pediatric primary care providers	No training details available for peer support group workshop	Usual care by pediatric primary care providers. It included child care visits and routine anticipatory guidance at 2 weeks, 1 month, 2, 4, and 6 months of age	a) Mean duration of BF: IG 22.7 weeks and CG 21.9 b) EBF at 6 months: IG 47% and CG 50%
Tylleskar, 2011 Burkina Faso, Uganda and South Africa	Burkina Faso and Uganda-Rural, South Africa- Mixed	Cluster RCT	Singleton birth without congenital malformation	Home visits by peer counselor mothers- one during antenatal and at least four during postnatal period, Burkina Faso: at weeks 1, 2, 4, 6, 16, and 20; Uganda and South Africa: weeks 1, 4, 7, and 10	Trained for 1 week on WHO breastfeeding support, HIV and infant feeding course	Usual care	a) EBF at 3 months: Burkina Faso-IG 79% and CG 35%, Uganda- IG 82% and CG 44%, South Africa- IG 10% and CG 6% b) EBF at 6 months: Burkina Faso-IG 73% and CG 22%, Uganda- IG 59% and CG 15%, South Africa- IG 2% and CG <1%
Wambach, 2011 USA	Urban	RCT	Primigravida women in second trimester pregnancy, age 15–18 years (middle adolescence)	A certified lactation consultant (also a registered nurse) and a trained peer counselor provided the intervention, composed of prenatal, in-hospital, and postnatal education and support through 4 weeks postpartum via conducting classes on breastfeeding, in- hospital visits and telephone counseling	Details not available	Two control groups: a) Attention control group (ACG) included same interventions as experimental group interventions except it did not focus on breastfeeding b) Usual care group (UCG) who received standard prenatal and postpartum care at their respective clinic	a) Any initiation of BF: IG 79%, ACG 66% and UCG 63% b) Initiation of EBF: IG 65%, ACG 68% and UCG 60% c) Formula supplementation at 3 weeks: IG 69&, ACG 70% and UCG 82%
Younes, 2015 Bangladesh	Rural	Quasi-experimental controlled before and after study (it was a follow up study of previous cluster RCT)	Women aged 15–49 years who had a child aged between 29 days and 5 years	Monthly meetings of womens' groups through a participatory learning and action cycle focusing on health issues of under-5 children including breast feeding, undernutrition, immunization etc. and facilitated by trained local woman. During earlier cluster RCT, they had focused on maternal and neonatal health	Facilitators received around 1 week training about participatory learning, communication, under 5 children's health problem, and community facilitation	Usual care	a) EBF at least 6 months: IG 81.4% and CG 50.6% b) Mean duration of EBF: IG 166.4 days and CG 109.8 days c) Minimum dietary diversity (percentage of children who received food from four or more food groups): IG 57.4% and CG 57.7%

We noted differences in the inclusion criteria of the study populations. The enrollment period for mothers varied among the studies, from any time during the antenatal period, either the first, second or third trimester of pregnancy to the postpartum period. Two studies exclusively included mothers who had low birth-weight infants [23, 24].

We also noted differences in the type of interventions, which included either one-on-one counseling or mothers' group meetings. Of 47 studies, 38 focused on one-on-one counseling with the remaining nine focusing on mothers' group meetings [6, 7, 17–19, 25–28]. One-on-one counseling was conducted by peer counselors during home visits, telephonic interactions, when the mother visited the antenatal clinic/hospital, or a combination of the above. Both, one-to-one counseling and mothers’ group meetings were conducted during antenatal, postpartum or both antenatal and postpartum periods. The number of contacts made by peer counselor or mothers' group meetings ranged from one to more than 10 visits. We noted differences in training periods for peer counselors and local facilitators of mothers’ group meetings. They ranged from a 4-hour session to over 6 months of classroom training and practice [9, 26]. We also noted that some peer counselors were paid employees [29–31], some received honorarium[32] or payment per visit [24, 33].We also found differences in the methods, frequency, and recall period in measuring EBF duration. Overall, the studies were heterogeneous and the sample sizes varied largely.

### Risk of bias in the selected studies

We noted differences in the risk of bias among the studies (S3 and S4 Tables). Of 41 RCTs and quasi-experimental studies, 22 were at high risk for the blinding of participants and personnel criteria, and 20 were classified as high risk due to the lack of outcome assessment blinding. Of six observational studies, two were assessed as high risk in terms of measurement of exposure [34, 35], two were assessed as high risk for blinding of outcome assessments [34, 36], three were assessed as high risk for incomplete outcome data [36–38], and two were assessed as high risk for selective outcome reporting [37, 38].

### Breastfeeding practices

#### a. Exclusive breastfeeding duration in low- and middle-income countries

**Fig 2:**
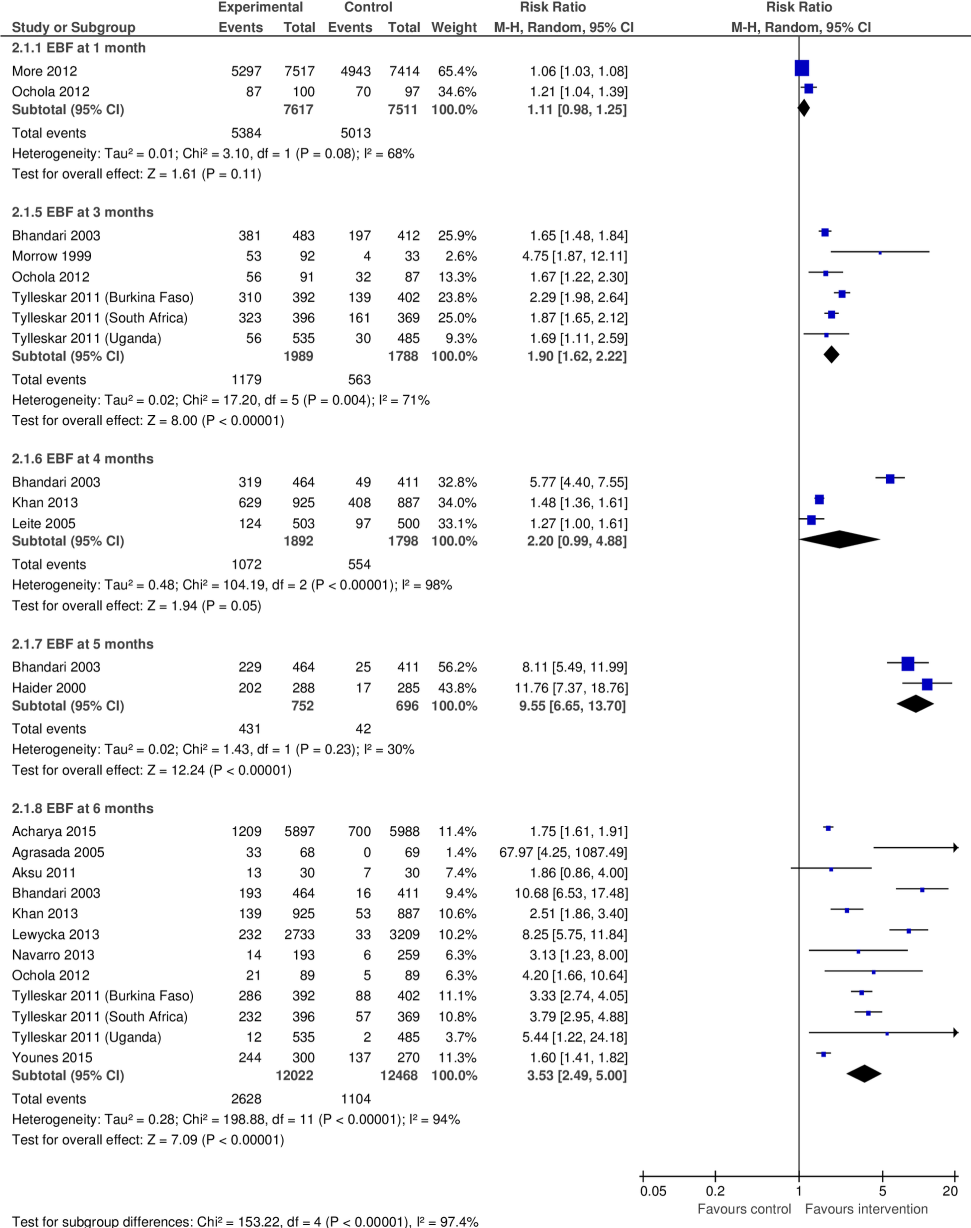
Pooled relative risk and 95% confidence intervals for the effect of community-based peer support for mothers on duration of exclusive breastfeeding in low- and middle income countries.

We included14 studies in the meta-analysis to measure the effect of community-based peer support for mothers on EBF duration in LMICs (Fig 2). Among them, two were quasi-experimental studies [6, 25] and the rest were RCTs. Furthermore, we conducted subgroup analyses by dividing all of these studies by different EBF duration.

#### Until 1 month

We included two studies in this subgroup analysis [28, 39] which comprised of 15,128 mothers. The pooled relative risk of continuing EBF until 1 month was 1.11 (95% CI: 0.98–1.25) for the intervention group versus the control (Fig 2). This estimate was characterized by substantial heterogeneity (I^2^ = 68%).

#### Until 3 months

We included four studies in this subgroup analysis [9, 20, 21, 39] which comprised of 3,777 mothers. The pooled relative risk of continuing EBF until 3 months was 1.90 (95% CI: 1.62–2.22) for the intervention group versus the control (Fig 2). This estimate was characterized by substantial heterogeneity (I^2^ = 71%).

#### Until 4 months

We included three studies in this subgroup analysis [22, 24, 40] which comprised of 3,690 mothers. The pooled relative risk of continuing EBF until 4 months was 2.20 (95% CI: 0.99–4.88) for the intervention group versus the control (Fig 2). The estimate was characterized by substantial heterogeneity (I^2^ = 98%).

#### Until 5 months

We included two studies in this subgroup analysis [22, 32] which comprised of 1,448 mothers. The pooled relative risk of continuing EBF until 5 months was 9.55 (95% CI: 6.65–13.70) for the intervention group versus the control (Fig 2). The estimate was characterized by low heterogeneity (I^2^ = 30%).

#### Until 6 months

We included 10 studies in this analysis [6, 7, 20, 22, 23, 25, 27, 39–41] which comprised of 24,490 mothers. The pooled relative risk of continuing EBF until 6 months was 3.53 (95% CI: 2.49–5.00) for the intervention group versus the control (Fig 2). This estimate had substantial heterogeneity (I^2^ = 94%).

#### b. Exclusive breastfeeding duration in high-income countries

**Fig 3:**
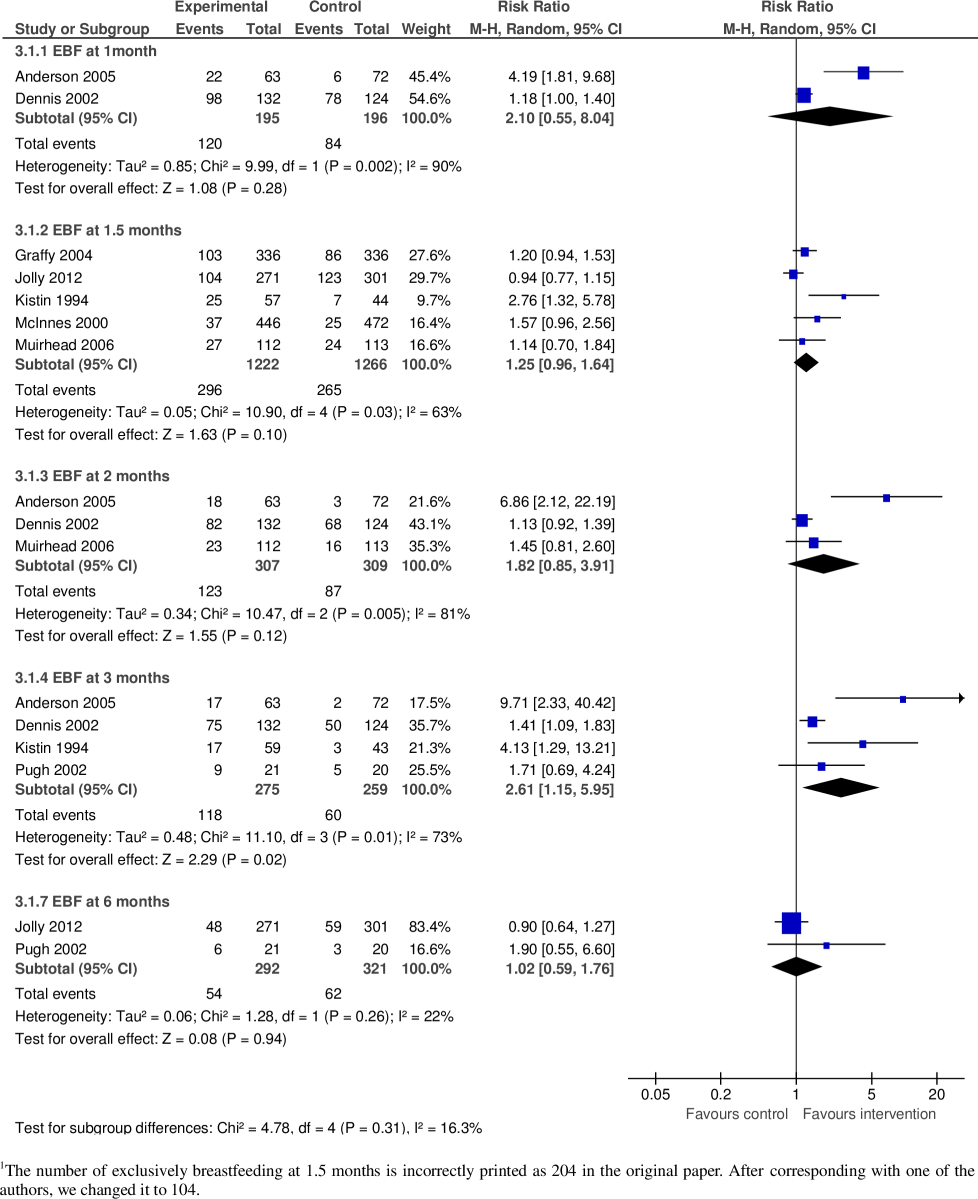
Pooled relative risk and 95% confidence intervals for the effect of community-based peer support for mothers on duration of exclusive breastfeeding in high income countries.

We included eight studies in this analysis to measure the effect of community-based peer support for mothers on EBF duration in high-income countries (Fig 3). Among them, two were quasi-experimental studies [42, 43] and the rest were RCTs. Furthermore, we conducted subgroup analyses by dividing all of these studies by EBF duration.

#### Until 1 month

We included two studies in this subgroup analysis [44, 45] which comprised of 391 mothers. The pooled relative risk of continuing EBF until 1 month was 2.10 (95% CI: 0.55–8.04) for the intervention group versus the control (Fig 3). This estimate was characterized by substantial heterogeneity (I^2^ = 90%).

#### Until 1.5months

We included five studies in this subgroup analysis [29, 33, 42, 43, 46] which comprised of 2,488 mothers. The pooled relative risk of continuing EBF until 1.5 months was 1.25 (95% CI: 0.96–1.64) for the intervention group versus the control (Fig 3). This estimate was characterized by substantial heterogeneity (I^2^ = 63%).

#### Until 2 months

We included three studies in this subgroup analysis [33, 44, 45] which comprised of 616mothers.The pooled relative risk of continuing EBF until 2 months was 1.82 (95% CI: 0.85–3.91) for the intervention group versus the control (Fig 3). This estimate was characterized by substantial heterogeneity (I^2^ = 81%).

#### Until 3 months

We included four studies in this subgroup analysis [43–45, 47] which comprised of 534 mothers. The pooled relative risk of continuing EBF until 3 months was 2.61 (95% CI: 1.15–5.95) for the intervention group versus the control (Fig 3). This estimate was characterized by substantial heterogeneity (I^2^ = 73%).

#### Until 6 months

We included two studies in this subgroup analysis [29, 47] which comprised of 292 mothers and the pooled relative risk of continuing EBF until 6 months was 1.02 (95% CI: 0.59–1.76) for the intervention group versus the control (Fig 3). This estimate had low heterogeneity (I^2^ = 22%).

#### c. Breastfeeding initiation within the first hour of life

**Fig 4:**
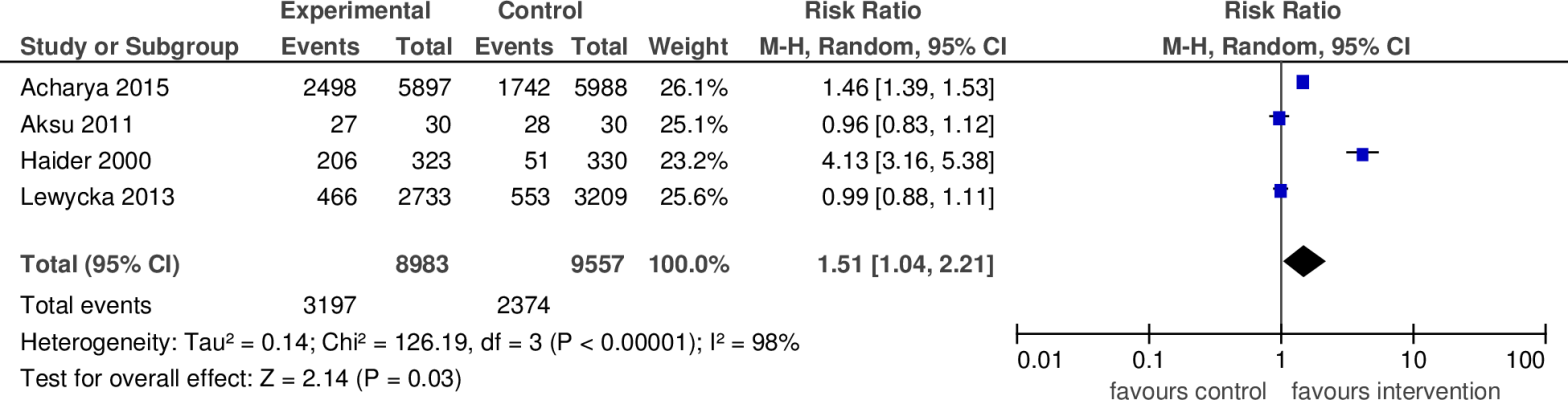
Pooled relative risk and 95% confidence intervals for the effect of community-based peer support for mothers on initiation of breastfeeding within the first hour of life in low- and middle income countries.

We included four studies in this analysis to measure the effect of community-based peer support for mothers on breastfeeding initiation within the first hour of life [7, 27, 32, 41]. All of the studies were conducted in LMICs. They included a total of 18,540 mothers and the pooled relative risk breastfeeding initiation was 1.51 (95% CI: 1.04–2.21) for the intervention group versus the control (Fig 4). This estimate had substantial heterogeneity (I^2^ = 98%).

#### d. Prelacteal feeding

**Fig 5:**
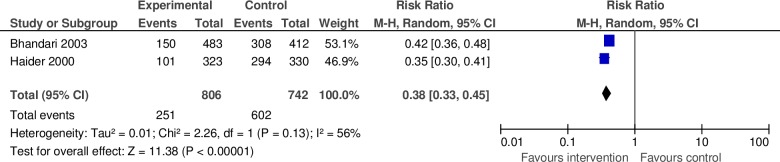
Pooled relative risk and 95% confidence intervals for the effect of community-based peer support for mothers on prelacteal feeding in low and middle income countries.

We included two studies in this analysis to measure the effect of community-based peer support for mothers on prelacteal feeding of infants [22, 32]. Both studies were conducted in LMICs. The analysis included a total of 1,548 participants and the pooled relative risk of prelacteal feeding was 0.38 (95% CI: 0.33–0.45) for the intervention group versus the control (Fig 5). The estimate was characterized by moderate heterogeneity (I^2^ = 56%).

#### e. Exclusive breastfeeding until 6 months (subgroup analysis by the type of interventions)

We conducted subgroup analyses by the type of intervention (one-on-one or group peer support) for the outcome of EBF until 6 months. We included 12 studies in this analysis. Among them, two were quasi-experimental studies [6, 25] and the rest were RCTs. Two studies were conducted in high-income countries [29, 47] and the rest in LMICs. We included eight studies in the subgroup one-on-one peer support [20, 22, 23, 29, 39–41, 47]. We included only four of the nine available studies for the subgroup of group peer support [6, 7, 25, 27]. Of the five excluded studies, two assessed a multifaceted intervention [17, 18], two did not report EBF at 6 months [26, 28], and one lacked a comparison group [19].

#### Interventions for one-on-one peer support for mothers

**Fig 6:**
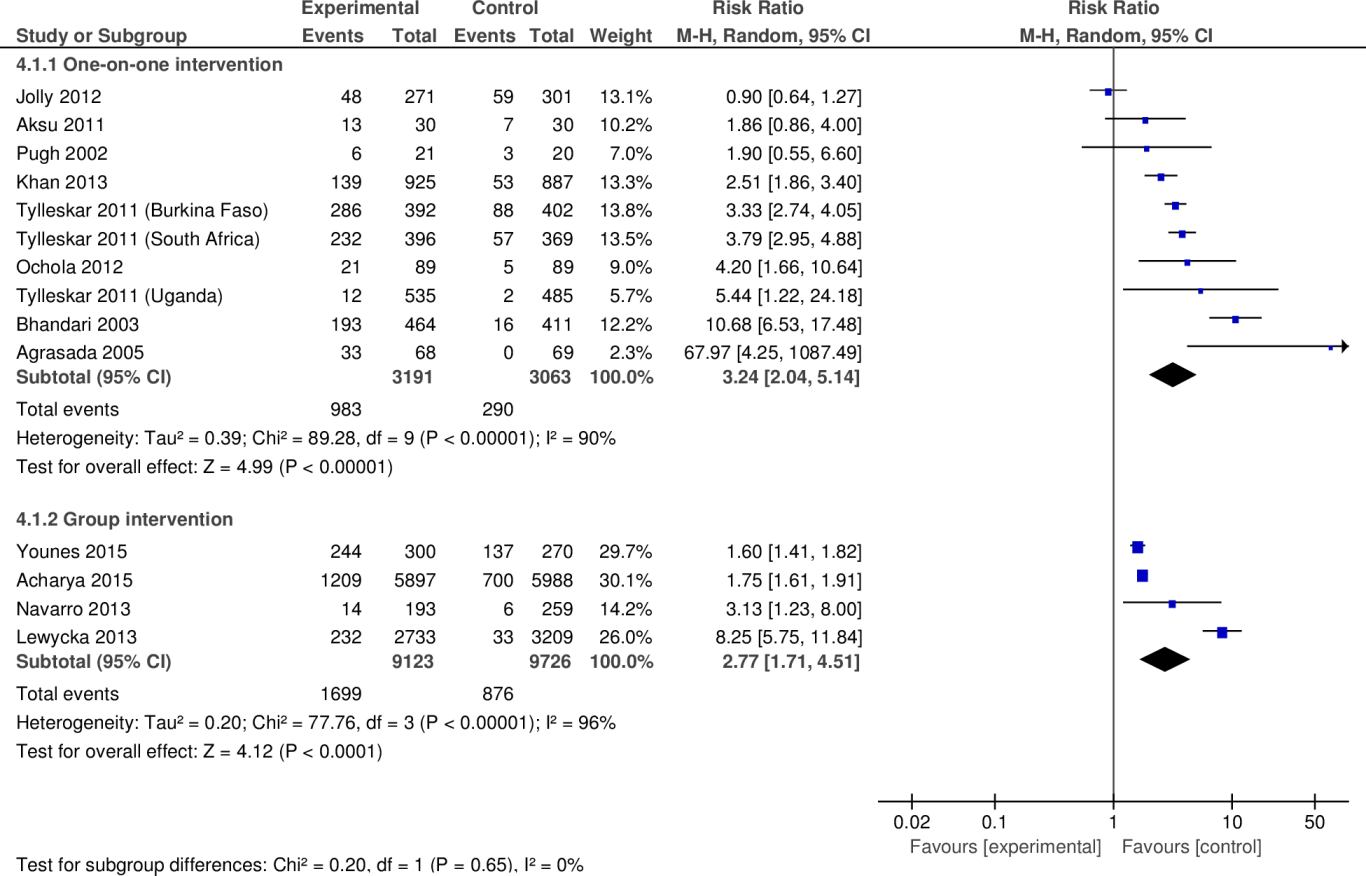
Pooled relative risk and confidence intervals for the effect of community-based peer support for mothers on exclusive breastfeeding at six months (subgroup analysis by type of the interventions).

The eight studies in this subgroup included 6,254 mothers. The pooled relative risk of continuing EBF until 6 months was 3.24 (95% CI: 2.04–5.14) for the intervention group versus the control (Fig 6). This estimate had substantial heterogeneity (I^2^ = 90%).

#### Interventions for group peer support for mothers

The four studies in this subgroup included 18,849 mothers. The pooled relative risk of continuing EBF until 6 months was 2.77 (95% CI: 1.71–4.51) for the intervention group versus the control (Fig 6). This estimate had substantial heterogeneity (I^2^ = 96%).

#### Sensitivity analysis

We conducted a sensitivity analyses to observe the effect of studies that had high risk of bias. We did this by comparing before- and after- meta-analysis results of the studies. However, we did not find any substantial differences or any publication biases.

## Discussion

This systematic review highlighted four major findings on the effectiveness of community-based peer support for mothers on their breastfeeding practices. First, community-based peer support for mothers can improve EBF duration. Moreover, mothers in LMICs continued EBF for a longer period than mothers in high-income countries. Second, such interventions can improve breastfeeding initiation within the first hour of life in LMICs. Finally, community-based peer support can reduce prelacteal feeding in LMICs.

Community-based peer support for mothers significantly increased EBF duration among mothers in both LMICs and high-income countries. In LMICs, mothers who received such support exclusively breastfed their infants until 3, 5 or 6 months compared to those who did not have such support. They even continued EBF until later months as suggested by the higher risk ratios in meta-analyses results at 5 and 6 months. In high-income countries, mothers who received such support exclusively breastfed their infants until 3 months compared to those without such support. We did not find any significant results proving effectiveness of peer support for EBF until the fifth and sixth months in high-income countries.

Mothers who received peer support in LMICs tended to exclusively breastfeed their infants for a longer period than such mothers in high-income countries. A separate systematic review also found that peer support had greater effect on EBF in LMICs than in high-income countries [10]. One possible explanation may be the higher social preferences for infant formula feeding in high-income countries[48]. Community-based peer support for mothers alone may be less effective in overcoming social barriers in those countries. In Scotland, social preferences for bottle feeding and aversion to public breastfeeding were likely factors responsible for the no-effect of peer support on EBF duration [42]. On the other hand, several factors create a favorable environment for EBF in LMICs, such as negative social attitudes towards infant formula, the high cost of infant formula, and the low prevalence of its commercial marketing [10, 49]. Therefore, support alone can be effective in increasing EBF duration in LMICs. In addition, mothers in high-income countries are likely to receive more advice and breastfeeding support from professional health workers[10]. Therefore, the definition of ‘usual care’ in high-income countries may be different from ‘usual care’ received in LMICs. This may be another reason for greater effectiveness of peer support in LMICs compared to that of high-income countries.

Mothers who received peer support in LMICs were more likely to initiate breastfeeding within the first hour of life than those who did not have such support. Moreover, peer support also decreased the risk of prelacteal feeding of newborns in LMICs. A separate systematic review also reported peer support as an effective intervention to promote breastfeeding initiation among low-income group mothers [50].

### Strengths and limitations

Our findings should be interpreted in line with the following limitations. First, we found differences in study populations, type of interventions, training methods, and outcome measurement methods, all of which may modify the effect of the interventions. This may also explain the source of substantial heterogeneity in some of our meta-analysis results. We addressed this by conducting subgroup analyses based on EBF duration and type of interventions (one-on-one or group peer support). Some of the subgroup analyses at different EBF follow-up time points were based on relatively smaller samples and may not have had the power to identify significant effects. We included quasi-experimental studies in the meta-analysis, which may have increased the risk of bias.

We were not able to assess the effect of community-based peer support for mothers when it was integrated into a multifaceted intervention. Only two studies included a multifaceted intervention. In the first study, the packaged intervention was the Integrated Management of Childhood Illness (IMCI) that significantly increased EBF duration [17]. The second study included peer support promotion as a part of its multifaceted intervention along with professional support [18]. However, we could not synthesize these results because packaged intervention components and the study area settings were different between these two studies. We did not analyze the effect of factors, such as health conditions of the mothers and their babies, and insufficiency of breast milk. These factors may have prevented mothers to exclusively breastfeed or to initiate breastfeeding within the first hour of delivery, even if they received peer support. We did not analyze the effect of alternative modes of breastfeeding such as breast milk expression by hand or pump. Lastly, we did not include any non-English language studies in this review.

Despite these limitations, this study is the first systematic review and meta-analysis to report the effectiveness of community-based peer support for mothers for different EBF durations ranging from 1 month to 6 months. Our findings may help policy makers to design low-cost and sustainable strategies to improve breastfeeding practices in locations where effective programming is lacking.

## Conclusions

Community-based peer support is effective in increasing EBF duration among mothers. Therefore, the mothers in LMICs continue EBF for much longer periods than the mothers in high-income countries. Such community-based peer support also enabled mothers to initiate breastfeeding early and avoid prelacteal feeding of newborns in LMICs. Moreover, mothers are more likely to exclusively breastfeed when they receive peer support one-on-one or through a mother’s group. Future studies are needed to explore sources of heterogeneity in such estimates and also examine the effect of a multifaceted intervention on breastfeeding practices.

## Supporting information

S1 Table PRISMA checklist. (DOCX)

S2 Table Excluded articles. (DOCX)

S3 Table Risk of bias assessments of RCTs and quasi-experimental studies. (DOCX)

S4 Table Risk of bias assessments of observational studies. (DOCX)

S1 File Systematic review protocol. (PDF)
