# Supplementary material for: Effectiveness of community-based peer support for mothers to improve their breastfeeding practices: A systematic review and meta-analysis
Source: PLoS One. 2017 May 16;12(5):e0177434. doi: 10.1371/journal.pone.0177434 (PMC5433692; doi:10.1371/journal.pone.0177434)
Supplement: S2 Table — (DOCX) [file pone.0177434.s002.docx]

**S2 Table- Excluded studies**

| **Study ID** | **Reasons for exclusion** |
| --- | --- |
| Adetugbo 1997 | Intervention by professional health workers |
| Anderson 2007 | Secondary study to Anderson 2005 |
| Bhandari 2003 | Secondary study to Bhandari 2003 |
| Dennis 2002 (September) | Secondary study to Dennis 2002 (January) |
| Feldens 2007 | Intervention- home visit by trained field worker. Outcome- Dental caries |
| Hotz 2005 | Intervention/training by field staff members |
| Kempenaar 2011 | Different outcome variables (Breastfeeding attitude and knowledge) |
| Khanal 2009 | Intervention not clear on one-to-one or group peer support |
| Kruske 2007 | Intervention by professional experts on child and family health |
| Laterra 2014 | Intervention not clear on one-to-one or group peer support |
| Mickens 2009 | Different outcome variable (Intention to breastfeed) |
| Mistry 2008 | Different outcome variables (Breastfeeding intention and attitude) |
| Nunes 2011 | Intervention by team of experts (doctors, nurses) |
| Oliveira 2012 | Intervention by team of experts (doctors, nurses, paediatricians) |
| Oliveira 2014 | Intervention by team of experts (doctors, nurses, paediatricians) |
| Penny 2005 | Intervention not clear on one-to-one or group peer support |
| Tarrant 2002 | Intervention not clear on one-to-one or group peer support |
