# Supplementary material for: Effectiveness of community-based peer support for mothers to improve their breastfeeding practices: A systematic review and meta-analysis
Source: PLoS One. 2017 May 16;12(5):e0177434. doi: 10.1371/journal.pone.0177434 (PMC5433692; doi:10.1371/journal.pone.0177434)
Supplement: S3 Table — (DOCX) [file pone.0177434.s003.docx]

**S3 Table- Risk of bias assessments of RCTs and quasi-experimental studies**


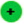
Indicates low risk of bias
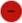
Indicates high risk of bias
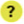
 Indicates unclear risk of bias

| Author | Sequence generation | Allocation concealment | Blinding of participants and personnel | Blinding of outcome assessments | Incomplete outcome data | Selective outcome reporting | Other potential bias |
| --- | --- | --- | --- | --- | --- | --- | --- |
| Acharya 2015 | 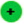 | 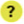 | 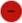 | 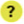 | 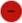 | 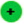 | 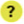 |
| Anderson 2005 | 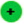 | 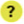 | 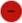 | 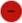 | 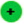 | 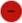 | 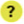 |
| Gross 1998 | 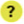 | 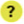 | 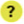 | 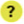 | 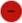 | 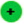 | 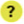 |
| Joly 2012 | 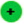 | 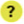 | 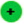 | 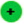 | 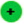 | 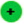 | 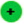 |
| Lewycka 2013 | 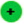 | 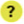 | 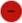 | 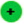 | 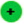 | 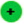 | 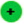 |
| Long 1995 | 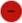 | 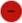 | 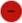 | 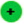 | 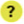 | 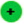 | 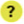 |
| Merewood 2006 | 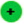 | 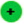 | 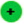 | 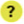 | 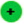 | 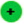 | 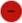 |
| Morrow 1999 | 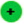 | 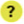 | 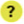 | 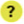 | 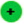 | 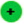 | 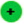 |
| Schafer 1998 | 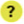 | 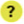 | 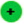 | 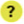 | 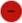 | 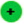 | 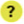 |
| Wambach 2011 | 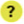 | 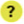 | 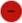 | 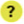 | 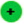 | 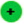 | 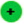 |
| Agrasada 2005 | 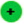 | 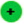 | 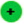 | 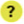 | 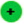 | 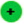 | 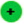 |
| Aksu 2011 | 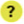 | 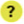 | 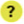 | 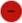 | 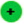 | 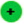 | 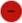 |
| Graffy 2004 | 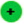 | 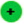 | 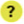 | 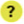 | 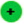 | 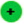 | 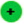 |
| Haider 2000 | 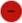 | 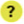 | 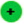 | 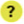 | 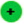 | 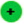 |  |
| Mclnnes2000 |  |  |  |  |  |  |  |
| Di Meglio 2010 |  |  |  |  |  |  |  |
| Navarro 2013 |  |  |  |  |  |  |  |
| Ochola 2013 |  |  |  |  |  |  |  |
| Pugh 2002 |  |  |  |  |  |  |  |
| Arifeen 2009 |  |  |  |  |  |  |  |
| Bhandari 2003 |  |  |  |  |  |  |  |
| Bhandari 2004 |  |  |  |  |  |  |  |
| Chapman 2004 |  |  |  |  |  |  |  |
| Chola 2013 |  |  |  |  |  |  |  |
| Edwards 2013 |  |  |  |  |  |  |  |
| Guldan 2000 |  |  |  |  |  |  |  |
| Hoddinott 2006 |  |  |  |  |  |  |  |
| Khan 2013 |  |  |  |  |  |  |  |
| Kisten 1994 |  |  |  |  |  |  |  |
| Kushwaha 2014 |  |  |  |  |  |  |  |
| More 2012 |  |  |  |  |  |  |  |
| Arlotti1998 |  |  |  |  |  |  |  |
| Dennis 2002 |  |  |  |  |  |  |  |
| Le Roux 2011 |  |  |  |  |  |  |  |
| Leite 2005 |  |  |  |  |  |  |  |
| Muirhead 2006 |  |  |  |  |  |  |  |
| Rempel 2012 |  |  |  |  |  |  |  |
| Shaw 1999 |  |  |  |  |  |  |  |
| Taveras 2011 |  |  |  |  |  |  |  |
| Tylleskar 2011 |  |  |  |  |  |  |  |
| Younes 2015 |  |  |  |  |  |  |  |
