# Supplementary material for: Effectiveness of community-based peer support for mothers to improve their breastfeeding practices: A systematic review and meta-analysis
Source: PLoS One. 2017 May 16;12(5):e0177434. doi: 10.1371/journal.pone.0177434 (PMC5433692; doi:10.1371/journal.pone.0177434)
Supplement: S4 Table — (DOCX) [file pone.0177434.s004.docx]

**S4 Table- Risk of bias assessments of observational studies**

| Author | Selection of participants | Confounding variables | Measurement of exposure | Blinding of outcome assessments | Incomplete outcome data | Selective outcome reporting |
| --- | --- | --- | --- | --- | --- | --- |
| Ahluwalia 2000 | 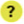 | 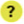 | 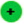 | 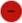 | 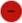 | 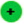 |
| Brown 2009 | 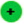 | 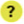 | 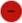 | 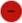 | 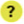 | 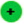 |
| Campbell 2014 | 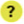 | 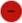 | 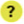 | 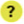 | 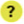 | 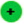 |
| Gross 2009 | 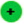 | 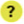 | 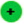 | 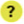 | 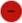 | 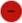 |
| Lovera 2010 | 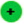 | 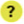 | 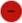 | 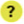 | 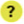 | 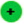 |
| Ingram 2013 | 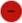 | 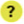 | 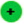 | 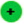 | 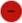 | 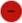 |


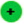
Indicates low risk of bias
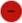
Indicates high risk of bias
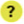
 Indicates unclear risk of bias
