# Supplementary material for: Effectiveness of community-based peer support for mothers to improve their breastfeeding practices: A systematic review and meta-analysis
Source: PLoS One. 2017 May 16;12(5):e0177434. doi: 10.1371/journal.pone.0177434 (PMC5433692; doi:10.1371/journal.pone.0177434)
Supplement: S1 File — (PDF) [file pone.0177434.s005.pdf]

UNIVERSITY of York
Centre for Reviews and Dissemination

NHS
National Institute for Health Research

PROSPERO International prospective register of systematic reviews

Effectiveness of participatory mothers' group interventions to improve child's nutritional status: a systematic review

Prakash Shakya, Mika Kunieda, Moe Miyaguchi, Hiroko Makino, Momoko Koyama, Sumi Dhakal, Sarju Rai, Su Sandy, Bruno Sunguya, Masamine Jimba

Citation

Prakash Shakya, Mika Kunieda, Moe Miyaguchi, Hiroko Makino, Momoko Koyama, Sumi Dhakal, Sarju Rai, Su Sandy, Bruno Sunguya, Masamine Jimba. Effectiveness of participatory mothers' group interventions to improve child's nutritional status: a systematic review. PROSPERO 2015:CRD42015019105 Available from http://www.crd.york.ac.uk/PROSPERO/display_record.asp?ID=CRD42015019105

Review question(s)

How effective are participatory mothers' group interventions in improving their feeding practices for children and their nutritional status?

Searches

We will search the following databases: PubMed/MEDLINE, Cochrane Library, CINAHL, Web of Science, SocIndex, PsycINFO. We will also hand search international organization databases and PhD thesis databases using the references of certain papers of interest. Searches will be limited to abstracts published in the English language and will be limited by year of publication from 1978 (Alma Ata declaration year, when community participation was first described as an important component of primary health care) to the end of March 2015.

Types of study to be included

Randomized controlled trial (RCT), non-randomized trials, cohort, quasi-experimental, observational, cross-sectional studies, and other comparative observational studies as well as multiple case studies and evaluation reports.
We will not include single case studies, letters, editorials, reviews, and books.

Condition or domain being studied

Child undernutrition contributes to about 45% of the 6.3 million child deaths every year (Liu et al., 2012, UNICEF, 2014). Undernutrition is caused by myriad of factors including poor feeding practices, childhood illnesses, and socio-demographic disadvantages (Black et al., 2008). Ensuring optimal infant and young child feeding (IYCF) practices is the key to improve nutritional status and thus promote the survival, growth and development of the child (Kushwaha et al., 2014).
Mothers are the primary caregivers of children in most communities. When mothers participate in group social activities, they are able to communicate with each other and exchange knowledge among themselves. Participatory mothers' group interventions have potential to improve IYCF practices and child's wellbeing (Lewycka et al., 2013). These interventions empower mothers through peer counseling and shared decision making (Younes et al., 2015). Evidence is scarce and inconsistent on the effectiveness of participatory mothers' group interventions on feeding practices and child's nutritional status. No systematic review has been conducted to examine such effectiveness and their effect on policy and practice. This systematic review aims to collate and summarize evidence on the effectiveness of mothers' groups in improving the feeding practices and nutritional status.

Participants/ population

Mothers of children under 5 years old

Intervention(s), exposure(s)

Inclusion criteria
1. Participatory interventions including peer nutrition counseling, shared decision making, grandmothers/elders to mother nutrition counseling OR
2. Nutrition- focused participatory interventions involving mothers' groups with mothers themselves as key drivers.
Exclusion criteria
1. Top-down nutrition interventions e.g. distribution of ready-to-use therapeutic foods (RUTF), blanket supplemental feeding programs, and cash transfer.

Comparator(s)/ control

We will include studies with and without control groups. If applicable, comparator will be comparable mothers' groups who have children under 5 years old and did not receive the interventions. We will also compare outcomes between pre and post intervention, where applicable.

Outcome(s)

Primary outcomes

Child nutritional status measured as underweight, wasting and stunting according to WHO Child Growth Standards median

a. Underweight: weight for age < -2 standard deviations (SD)
b. Stunting: height for age < -2 SD
c. Wasting: weight for height < -2 SD

Secondary outcomes

Feeding practices for children measured through

a. Breast feeding (exclusive breastfeeding and duration): number of months
b. Initiation of complementary feeding: age in months
c. Continued breast feeding duration (number of months)
d. Feeding frequency: number of meals per day
e. Step up period during complementary feeding: age in months
f. Dietary diversity: measured as the number of types of foods fed to the child in one day

Data extraction, (selection and coding)

Paired researchers will conduct the literature search and review the articles for eligibility. We will discuss disagreements on eligibility of study until a consensus is reached. If required, we will consult the supervisor for final decision.

Risk of bias (quality) assessment

We will assess each selected study for selection bias, performance bias, detection bias, attrition bias and reporting bias using GRADE criteria.

Strategy for data synthesis

We will conduct a descriptive analysis of individual studies according to the type of intervention, duration, outcome, quality and risk of bias. We will analyze the effectiveness of intervention, based on the nature of reported outcomes. If we find enough studies with quality data, we will conduct a meta-analysis to examine the effectiveness of mothers' group-based empowerment interventions

Analysis of subgroups or subsets

None planned at this time

Dissemination plans

The review will be published in a peer reviewed scientific journal

Contact details for further information

Dr Shakya
Department of Community and Global Health, Graduate School of Medicine
The University of Tokyo, 7-3-1, Hongo, Bunkyo-ku, Tokyo, 113-0033 Japan
canvas_76@yahoo.com

Organisational affiliation of the review

Department of Community and Global Health, The University of Tokyo
http://www.ich.m.u-tokyo.ac.jp/en/index.html

Review team

Dr Prakash Shakya, Department of Community and Global Health, The University of Tokyo
Ms Mika Kunieda, Department of Community and Global Health, The University of Tokyo
Ms Moe Miyaguchi, Department of Community and Global Health, The University of Tokyo
Ms Hiroko Makino, Department of Community and Global Health, The University of Tokyo
Ms Momoko Koyama, Department of Community and Global Health, The University of Tokyo
Ms Sumi Dhakal, Department of Community and Global Health, The University of Tokyo
Mr Sarju Rai, Department of Community and Global Health, The University of Tokyo
Dr Su Sandy, Department of Community and Global Health, The University of Tokyo
Dr Bruno Sunguya, Department of Community and Global Health, The University of Tokyo
Professor Masamine Jimba, Department of Community and Global Health, The University of Tokyo

Anticipated or actual start date

01 April 2015

Anticipated completion date

15 May 2015

Funding sources/sponsors

Department of Community and Global Health, Graduate School of medicine, The University of Tokyo, Japan

Conflicts of interest

None known

Language

English

Country
Japan

Subject index terms status
Subject indexing assigned by CRD

Subject index terms
Child; Child Nutritional Physiological Phenomena; Humans; Mothers; Nutritional Status

Stage of review
Ongoing

Date of registration in PROSPERO
01 April 2015

Date of publication of this revision
01 April 2015

DOI
10.15124/CRD42015019105

Stage of review at time of this submission	Started	Completed
Preliminary searches	Yes	No
Piloting of the study selection process	No	No
Formal screening of search results against eligibility criteria	No	No
Data extraction	No	No
Risk of bias (quality) assessment	No	No
Data analysis	No	No

PROSPERO
This information has been provided by the named contact for this review. CRD has accepted this information in good faith and registered the review in PROSPERO. CRD bears no responsibility or liability for the content of this registration record, any associated files or external websites.
